# Management of an obstructed recurrent inguinal hernia using a hybrid method: a case report

**DOI:** 10.1186/s12893-021-01069-7

**Published:** 2021-01-21

**Authors:** Yuka Ooe, Naoki Horikawa, Shohei Miyanaga, Ryosuke Kobiyama, Yurika Iida, Ayako Kanamoto, Wataru Fukushima, Kazuhisa Yabushita

**Affiliations:** Department of Surgery, Takaoka City Hospital, 4-1 Takaramachi, Takaoka, Toyama 933-8550 Japan

**Keywords:** Recurrent inguinal hernia, Incarcerated inguinal hernia, Hybrid surgery

## Abstract

**Background:**

For recurrent incarcerated and strangulated hernias, the optimal treatment strategy for each case is needed.

**Case presentation:**

The study patient was a 70-year-old man. TAPP repair was performed for a left inguinal hernia (JHS Classification II-1) 7 years earlier. The patient experienced transient pain and swelling of the left inguinal region for 5 months and visited our emergency department for abdominal pain and vomiting. A CT scan showed a recurrent left inguinal hernia and small bowel incarceration, and emergency surgery was performed. Laparoscopic observation of the abdominal cavity revealed recurrent left inguinal hernia (Rec II-1) with small bowel incarceration. The small bowel was reduced after pneumoperitoneum, and no findings suggested intestinal tract necrosis. Adhesions around the herniated sac were dissected using an extraperitoneal approach and then shifted to mesh plug repair. No perioperative complications or hernia recurrence were observed in the 10 months after the surgery.

**Conclusions:**

This report describes a novel, successful surgical treatment for a recurrent incarcerated hernia. In our patient, we could easily perform dissection and understand the positional relationship by hybrid surgery using the TEP method. Additionally, in patients with incarcerated hernias, we believe that performing hybrid surgery by combining the TEP method would be useful because bowel dilation caused by intestinal obstruction would not disturb the operative field.

## Background

Recurrent inguinal hernia is difficult to understand anatomically, and its repair is often challenging [1]. Therefore, several guidelines [2–4] propose that repeat laparoscopic repair procedures should be performed by a surgeon with sufficient procedural skill.

Furthermore, in the treatment of incarcerated and strangulated inguinal hernias, an open approach is recommended because no other additional skin incision is needed when performing intestinal resection. However, various judgments should be made for each case.

We successfully treated a patient with recurrent incarcerated hernia following repair with the transabdominal preperitoneal (TAPP) approach with hybrid surgery combining the extraperitoneal approach with mesh plug repair.

## Case presentation

### Patient

A man in his 70 s.

When he was in his 60 s, the patient underwent surgery for a left inguinal hernia [TAPP method, Japanese Hernia Society (JHS) classification [2] II-1, Bard^®^ 3D MAX Light, M size].

He visited the emergency outpatient services of our hospital due to abdominal pain and vomiting 2 h prior. His abdomen was swollen and tense. In the left inguinal region, tender golf ball-sized swelling was noted. Abdominal and pelvic computed tomography (CT) findings showed a recurrent left inguinal hernia with complications of small intestine incarceration and obstruction. Ascites was observed within the hernia sac.

Upon suspicion of incarcerated hernia, manual reduction was attempted. However, reduction could not be achieved. Emergency surgery was adopted as the treatment policy.

### Surgical findings

**Fig. 1:**
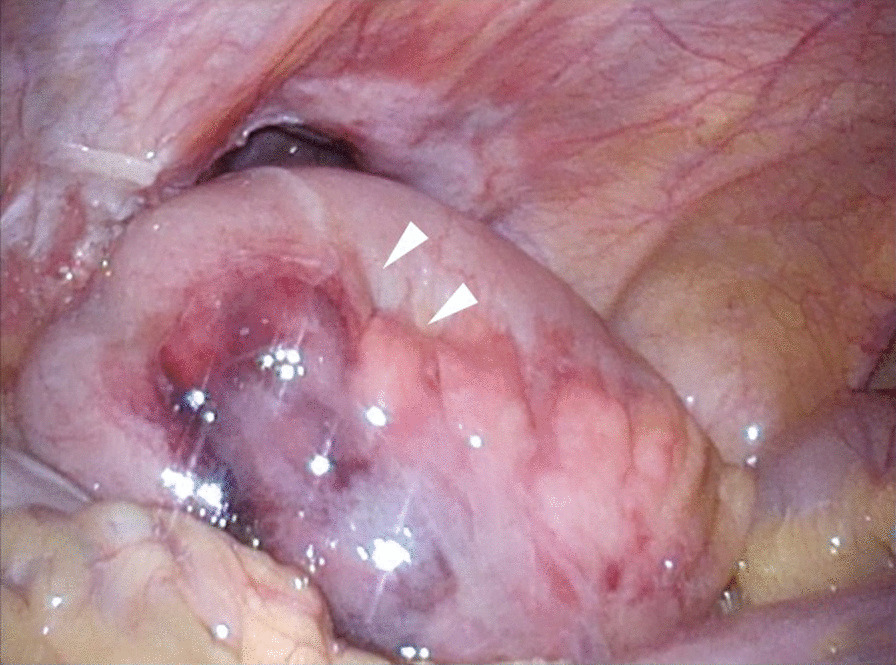
Intraoperative findings. Small intestine is released during pneumoperitoneum. A mesenteric hematoma is observed (arrow), but no findings of necrotic small intestine are noted

**Fig. 2:**
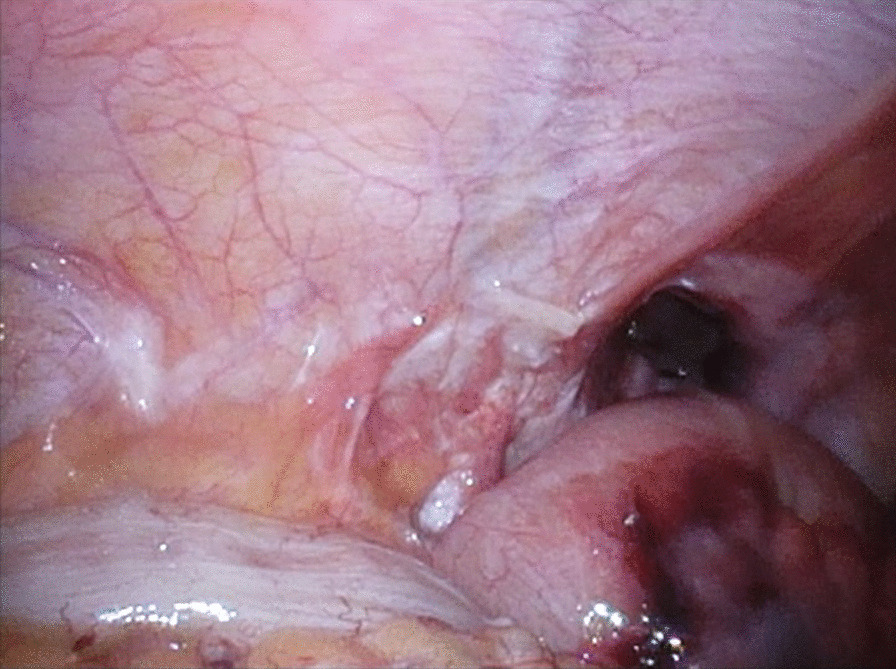
Left recurrent inguinal hernia with mesh displaced laterally

**Fig. 3:**
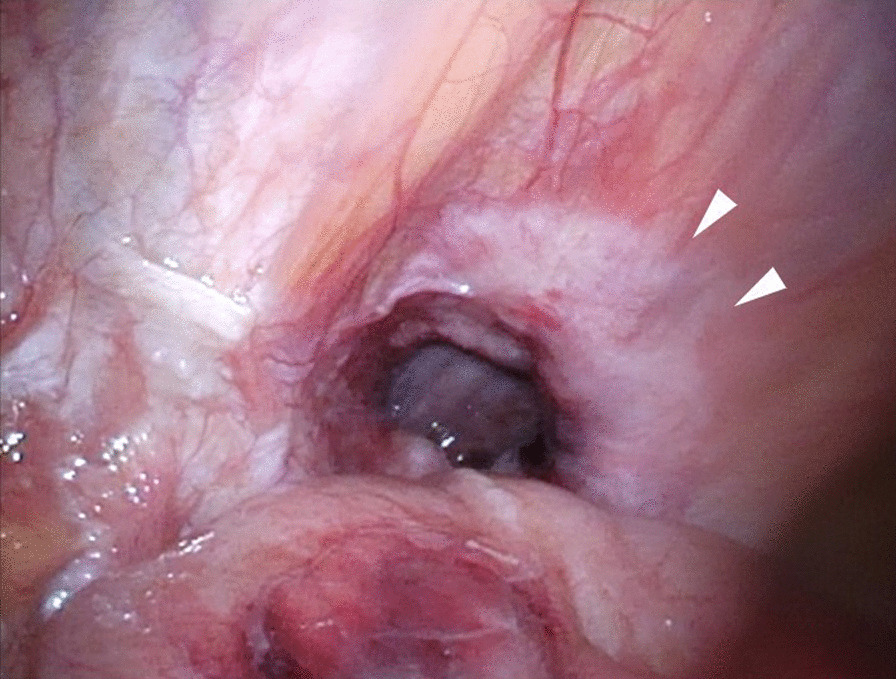
Scar tissue around hernia ring (arrow)

**Fig. 4:**
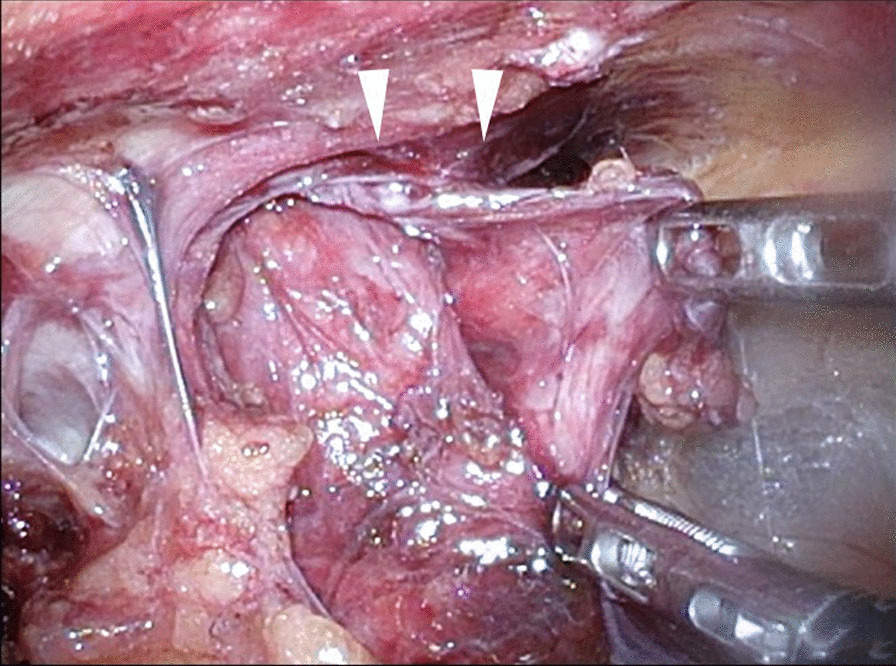
Preperitoneal space. Dissection of adhesion around the sac. The hernia sac was observed at the inguinal orifice (arrow)

**Fig. 5:**
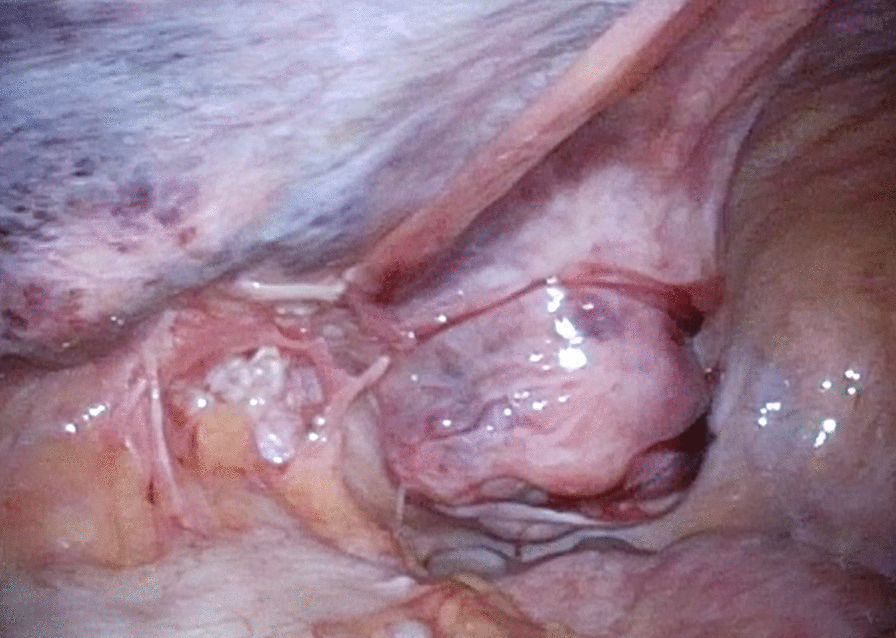
Re-examination of the intraperitoneal space. The hernia was repaired and no findings suggested strangulation in the bowel

**Fig. 6:**
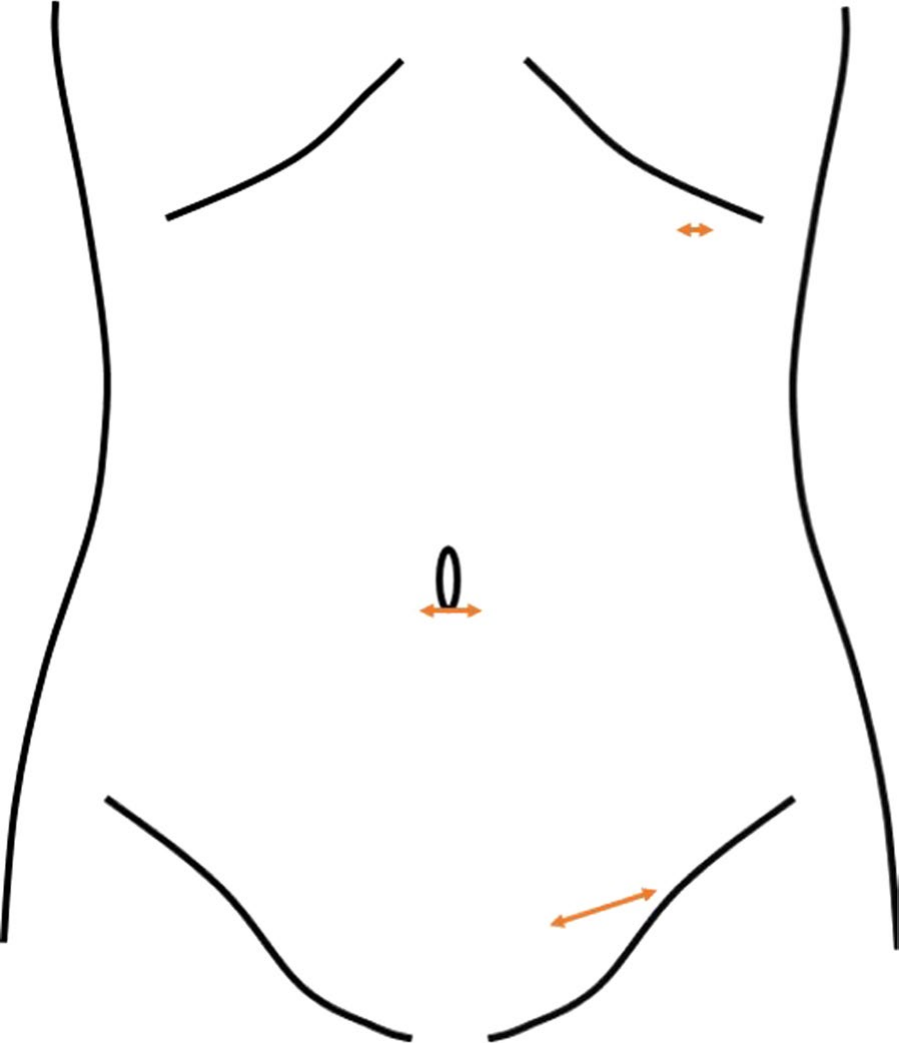
Illustration of the postoperative wound (created by authors)

We judged that it was risky to insert the first port on the navel. Referring to the CT scan, we inserted the first port in the upper left abdomen for laparoscopy. Laparoscopic observation revealed the recurrence of left inguinal hernia (JHS classification Rec II-1), incarceration of the small intestine, and general dilatation of the bowel due to intestinal obstruction. Following pneumoperitoneum, the incarcerated small intestine spontaneously reduced. Mild hematoma was observed in the mesentery of the incarcerated bowel; however, there were no clear findings that suggested strangulation (Fig. 1). The mesh of the initial surgery was found to extend from near the root of inferior epigastric vessels to the medial umbilical fold (Fig. 2). The hernia orifice was found in Hesselbach’s triangle, and particularly severe scarring was noted on the medial side of the hernia orifice (Fig. 3). We assumed that the recurrence occurred as the first mesh was corrugated and shifted. Considering the difficulty involved in ensuring the visual field due to bowel dilatation using the TAPP method, we dissected the adhesions surrounding the hernia sac as much as possible using the TEP method (Fig. 4). In the extraperitoneal space, there was adhesions especially at the inner side of the hernia orifice, it was slightly difficult to treat adhesions at this site. Thereafter, we switched to mesh plug repair. The hernia sac could be easily identified and treated with Bard^®^ Mesh Plug and an onlay patch. Upon re-examination of the intraperitoneal space, we confirmed that the hernia was repaired (Fig. 5), and no findings suggested strangulation in the bowel. The operative duration was 3 h and 40 min with minimal blood loss. The postoperative wound is presented in Fig. 6.

### Postoperative progress

We did not observe any perioperative complications, and the subject was stable enough to be discharged on postoperative day 6. After rehabilitation, the subject was discharged on postoperative day 11. At the time of writing this report, at 10 months postoperatively, no signs of recurrence or infection were observed.

## Discussion and conclusions

With regard to surgical procedures for recurrent hernia, few high-quality reports have recommended specific procedures. The presence or absence of preperitoneal detachment with prior surgery has the greatest impact on the selection of surgical procedure for recurrent hernia. The World Guidelines for Groin Hernia Management published as a draft by the HerniaSurge Group recommend anterior repair for recurrence following posterior repair, including laparoscopic surgery. Moreover, several guidelines also suggest that experienced practitioners select the surgical procedure based on comorbidities, form of recurrence and practitioner skill level [2–5].

The advantage of using laparoscopy for recurrent inguinal hernia is that observation of the inguinal region with laparoscopy provides useful information on recurrence characteristics (e.g., the location of the hernia orifice and the previous mesh). It is important to confirm the dislocation of the previously placed mesh, the positional relationship of the mesh to the hernia orifice, and the degree of adhesion to prevent re-recurrence [6]. Furthermore, observation after repair makes it possible to confirm the adequacy of deployment of the newly inserted mesh [7]. However, this information cannot be obtained enough using intraperitoneal observation alone. Therefore, we adopted the preperitoneal approach (i.e., TEP repair). We dissected around the hernia sac as much as possible with preservation of the vasculature with the TEP technique. We believe it is advantageous when switching to mesh plug repair because it enables identification and dissection of the hernia sac to be performed safely and easily.

Factors that affect the selection of surgical procedures for hernias include the presence or absence of bowel incarceration and strangulation. Evidence in support of laparoscopic surgery for patients with incarcerated and strangulated hernias is limited.

Even if the incarcerated hernia is spontaneously reduced, intraperitoneal observation is recommended to assess the incarcerated organ [8]. At present, there are no established treatment methods for strangulated hernia. In patients with irreversible blood flow impairment in the incarcerated bowel and those requiring bowel resection and anastomosis, the approach and mesh use remain controversial [9]. To our knowledge, no RCTs have compared the two procedures, TAPP and TEP repair in incarcerated or strangulated hernia. We believe that TEP repair is useful because it enables the separation of the clean operative field and contaminated operative field, and even if concurrent bowel obstruction and the space within the peritoneum is limited, surgery can be performed easily with a relatively good visual field [10].

For recurrent incarcerated and strangulated hernias, the optimal treatment should be selected for each case, such as the details of previous surgery, skill level of the practitioner, and general condition of the patient. Based on our experience, we believe that performing concurrent TEP repair in the hybrid method is useful for dissecting around the hernia sac and reduces the risk of repeat recurrence. For cases of incarcerated and strangulated hernia, we also consider the method to be useful for securing the visual field and for isolating the noncontaminated area when performing contaminated surgery.

## Data Availability

Not applicable.

## Abbreviations

TEP: Totally extraperitoneal
TAPP: Transabdominal preperitoneal
JHS: Japanese Hernia Society
CT: Computed tomography
